# Strategies for Improved Intra-arterial Treatments Targeting Brain Tumors: a Systematic Review

**DOI:** 10.3389/fonc.2020.01443

**Published:** 2020-08-26

**Authors:** Rui Huang, Johannes Boltze, Shen Li

**Affiliations:** ^1^Department of Neurology, Dalian Municipal Central Hospital Affiliated With Dalian Medical University, Dalian, China; ^2^School of Life Sciences, University of Warwick, Coventry, United Kingdom

**Keywords:** brain tumor, intra-arterial, targeted therapy, chemotherapy, blood–brain barrier, nanoparticles, superselective cerebral infusion, imaging

## Abstract

Conventional treatments for brain tumors relying on surgery, radiation, and systemic chemotherapy are often associated with high recurrence and poor prognosis. In recent decades, intra-arterial administration of anti-cancer drugs has been considered a suitable alternative drug delivery route to intravenous and oral administration. Intra-arterial administration is believed to offer increasing drug responses by primary and metastatic brain tumors, and to be associated with better median overall survival. By directly injecting therapeutic agents into carotid or vertebral artery, intra-arterial administration rapidly increases intra-tumoral drug concentration but lowers systemic exposure. However, unexpected vascular or neural toxicity has questioned the therapeutic safety of intra-arterial drug administration and limits its widespread clinical application. Therefore, improving targeting and accuracy of intra-arterial administration has become a major research focus. This systematic review categorizes strategies for optimizing intra-arterial administration into five categories: (1) transient blood-brain barrier (BBB)/blood-tumor barrier (BTB) disruption, (2) regional cerebral hypoperfusion for peritumoral hemodynamic changes, (3) superselective endovascular intervention, (4) high-resolution imaging techniques, and (5) others such as cell and gene therapy. We summarize and discuss both preclinical and clinical research, focusing on advantages and disadvantages of different treatment strategies for a variety of cerebral tumor types.

## Introduction

Brain tumors are a large class of heterogeneous neoplasms, generally classified as benign or malignant tumors. It is widely believed that uncontrolled proliferation and tissue infiltration of dedifferentiated cells caused by harmful chemical, physical, and biological exposures are the main causes of brain tumor malignancy (1). Non-malignant brain tumors also can pose a much higher risk than elsewhere in the body, in particular when situated in areas hard to reach by surgical interventions. According to the latest statistics from the United States Central Brain Tumor Registry, the average annual incidence of brain and other central nervous system (CNS) tumors (malignant and non-malignant) after age adjustment was 23.03 per 100,000 people (2). Moreover, a worldwide meta-analysis reported that the total incidence of primary brain tumors was 10.82 per 100,000 person-years, with an estimated range of 0.01 (pineal tumors) to 25.95 (all primary brain tumors) per 100,000 people (1). As of 2011, several population-based studies indicated that the incidence of metastatic brain tumors was 7–14 per 100,000 people (3), thus accounting for 2% of all cancers and 12.1% of metastatic diseases (4). The brain tumor incidence is related to a variety of factors, such as the type, location, and grade of a primary tumor in case of metastases, as well as the age, gender, ethnicity, and risk factor exposure of the patients (1, 5). For example, primary brain tumors are most common in children aged 0–14 years (2, 6) and include hair cell astrocytoma, embryonic tumors, and malignant gliomas (6). Meningiomas have the highest incidence of all adult primary brain tumors (3), followed by malignant gliomas and pituitary tumors (3, 4). It is noteworthy that brain metastases are more common in adults, with lung cancer, breast cancer, and melanoma showing the highest rates (3, 4).

Brain tumors are relatively rare compared to other neurological diseases, and complex pathogenesis of some brain tumor types (especially malignant gliomas) often renders existing treatment strategies ineffective in prolonging survival time. Thus, brain tumors are the most life-threatening cancers in humans (7). The traditional treatment of brain tumors is the combination of surgery, radio- and chemotherapy (8). Surgery targets at the removal of tumor tissue. Radio- and chemotherapy as primary or adjuvant treatments have a positive effect on improving the survival rate of selected patients (9) and can reduce tumor mass prior to surgery. Anti-tumor effects are not only based on drug dose and tumor sensitivity but also related to the route of drug delivery. In general, drug binding to plasma proteins or lipids in the peripheral circulation and the target organ metabolism affect therapeutic effects after systemic administration (10). Systemic exposure in non-targeted organs caused by intravenous and oral administration are also an important factor restricting long-term use of chemotherapy drugs (11).

Intra-arterial administration was first described for the treatment of brain tumors in the 1950s (12). In comparison to intravenous delivery, intra-arterial administration increases intra-tumor drug concentration and accelerates systemic clearance (13). Up to now, intra-arterial administration has been shown to exert positive effects on recurrent or progressive malignant glioblastomas (14), retinoblastomas (15), and primary CNS lymphomas (16). However, severe vascular toxicity and neurotoxicity have been reported in several clinical studies. For example, patients with primary glioblastomas or anaplastic astrocytomas receiving intra-arterial cisplatin often experience significant loss in high-frequency hearing (17, 18). Moreover, patients with newly diagnosed or recurrent malignant gliomas or other brain tumors suffer from decreased visual acuity and irreversible encephalopathy (e.g., cerebral edema and leukoencephalopathy) when receiving intra-arterial carmustine 1,3-Bis(2-chloroethyl)-1-nitrosourea (BCNU) (19, 20). Myelosuppression mostly occurs in patients with primary CNS lymphomas who have received intra-arterial nitrosourea 1-(4-amino-2-methyl-5-pyrimidinyl)methyl-3-(2-chloroethyl)-3-nitrosourea (ACNU) and radiotherapy (21). Myelosuppression is also the most common side effect of intra-arterial cisplatin combined with oral etoposide in the treatment of recurrent malignant gliomas (22). Other relatively common complications include nausea, vomiting, thrombocytopenia, seizures, ocular pain, headache, intratumoral hemorrhage, transient cerebral ischemia, granulocytopenia, nephrotoxicity, and vasospasm (19, 21, 23–27). Radiotherapy before intra-arterial administration may contribute to white matter necrosis (20, 28). Moreover, technical (e.g., perfusion strategy and artery selection) and pharmacological factors (e.g., drug dosage and compatibility) can affect safety, accuracy, and efficacy of intra-arterial administration (29–31) and have not been discussed in detail so far.

On the other hand, a number of strategies to improve the therapeutic effects of intra-arterial administration for the treatment of brain tumors have been suggested. This systematic review summarizes the five most important ones. The first option is blood–brain barrier (BBB)/blood-tumor barrier (BTB) disruption using chemical reagents (such as mannitol, bradykinin, or alkylglycerols) or penetration drug carriers (such as liposomes, micelles, cell-penetrating peptides) to increase the direct entry of drugs into tumor and brain tissues. The second option is intra-arterial injection during transient cerebral hypoperfusion (IA-TCH) or flow arrest (IA-FA). This increases local plasma drug concentration and exposure time by reducing dilution, absorption, and contact with blood components. The third option are microcatheters designed to allow superselective intra-arterial cerebral infusion (SIACI) into the tumor-feeding arteries to reduce neurotoxic side effects while achieving well-targeted drug delivery. The fourth option is to combine imaging techniques, such as MRI, CT, X-ray, SPECT, and PET with intra-arterial infusion of labeled therapeutic agents, to monitor delivery and accumulation in the tumor and brain parenchyma. The fifth option is to use advanced techniques including intra-arterial infusion of gene-edited viruses or cells to achieve targeted molecular or cell therapy for brain tumors. Next to providing an overview, this review also discusses advantages and limitations of these different strategies that became obvious in preclinical and clinical research.

## Methodological Approach

Literature search and evaluation were conducted according to the Preferred Reporting Items for Systematic Reviews and Meta-Analysis (PRISMA) standard. No ex-ante protocol was used.

A systematic literature search was conducted in three databases: EMBASE, PubMed, and Web of Science. The original search date was October 22, 2019 (*n* = 1,835) and updated on June 30, 2020 (*n* = 1,855). One more study was included during the updated search. The publication time in EMBASE was 1963–2020, PubMed 1962–2020, and Web of Science 1946–2020. A keyword-based search strategy was applied as follows: In EMBASE, the keyword terms “brain tumor” and “intraarterial drug administration” of EMTREE dictionary were used. The MeSH-defined keywords “Brain Neoplasms” and “Injections, Intra-Arterial” or “Infusions, Intra-Arterial” were used in PubMed searches, while Web of Science searches were conducted with “Brain tumor” and “Intra arterial.” In addition, other eligible publications selected from the list of references in the included literature were used to supplement the search results. Only peer-reviewed articles published in English were included. The included literature should focus on strategies that aimed at improving the therapeutic effect of intra-arterial treatment. Therefore, articles that simply evaluated the therapeutic effects of intra-arterial administration but without comparing them to alternate approaches were excluded (*n* = 105). We also excluded conference abstracts (*n* = 10), papers without available full-text (*n* = 3), papers not reporting brain tumor treatment (*n* = 21), and *in-vitro* studies (*n* = 5), as well as studies not reporting intra-arterial treatment (*n* = 4) or being focused on embolization only (*n* = 5). Reviews were also excluded (*n* = 28).

## Results

### Data Set

**Figure 1:**
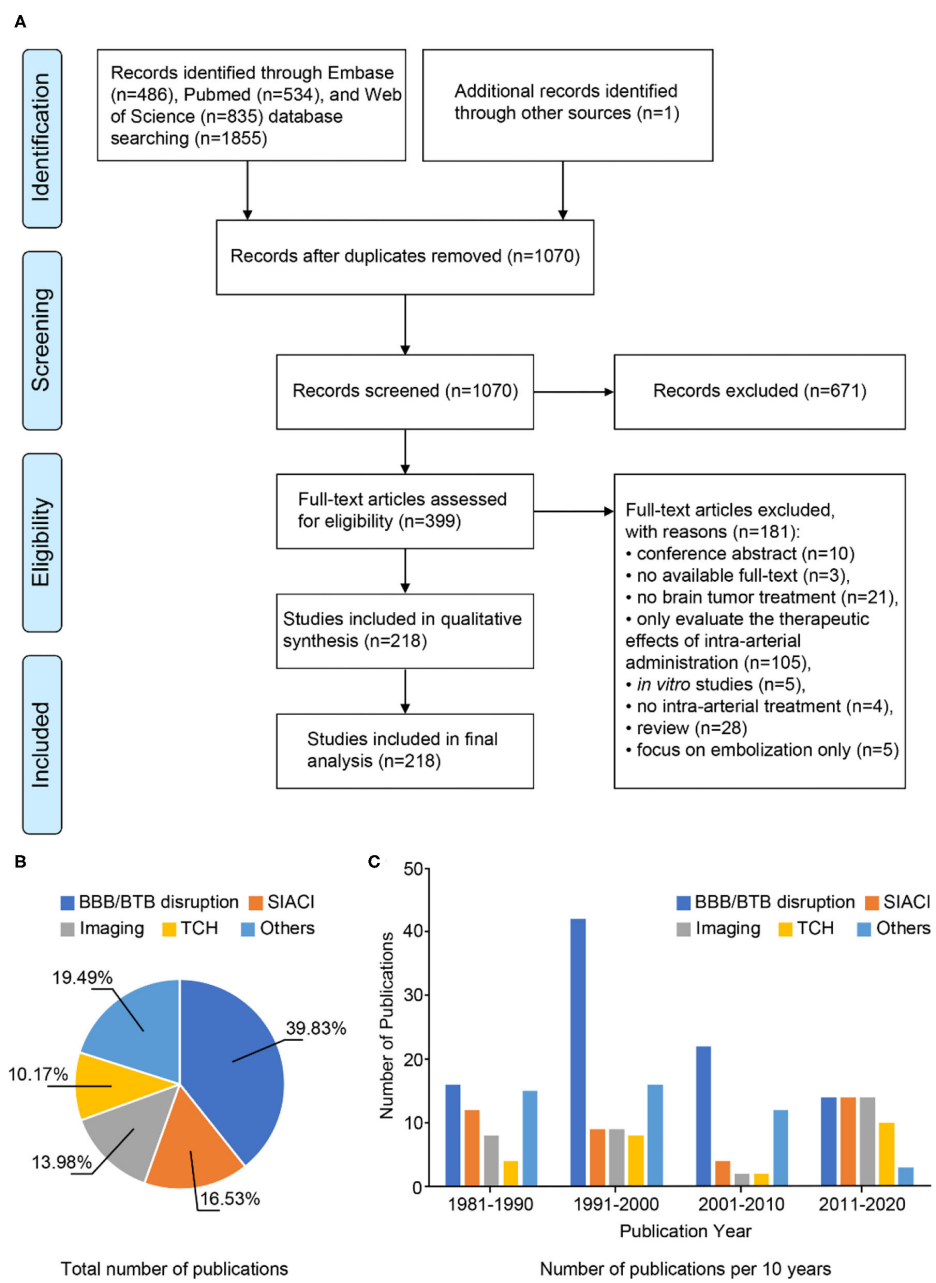
Methodological approach. **(A)** PRISMA flow diagram. **(B)** Pie chart of the total number of publications between 1981 and 2020. **(C)** Temporal trend graph of the numbers of publications for different strategies. Since 1981, the numbers of articles on BBB/BTB disruption published in consecutive decades are 16, 42, 22, and 14, respectively. The numbers of articles on SIACI published in consecutive decades are 12, 9, 4, and 14, respectively. The numbers of articles on imaging published in consecutive decades are 8, 9, 2, and 14, respectively. The numbers of articles on TCH published in consecutive decades are 4, 8, 2, and 10, respectively. The numbers of articles on other strategies published in consecutive decades are 15, 16, 12, and 3, respectively.

A total of 1,855 articles were retrieved from EMBASE (*n* = 486), PubMed (*n* = 534), and Web of Science databases (*n* = 835). By screening the reference lists of the included papers, one more publication was added to the search results, resulting in a total of 1,856 articles. Screening for duplicates and their removal resulted in a total of 1,070 articles. We next screened titles and abstracts, obtaining 399 articles not meeting any exclusion criterion. Finally, 218 articles published between 1981 and 2020 were included after full-text evaluation (Figure 1A), what allowed us analyzing literature output in the previous four decades.

We then grouped articles according to the strategies described for optimizing intra-arterial administration of therapeutic agents to brain tumors. There were 94 studies focusing on BBB/BTB disruption, 24 on TCH, 39 on SIACI, 33 on imaging-guided approaches, and 46 on other therapeutic agents and methods (Figure 1B).

As shown in Figure 1C, research on optimization strategies changes with the development of new technologies. The majority of studies focused on BBB disruption and were most frequently published 1991–2000 (*n* = 42). Numbers of studies focusing on BBB disruption gradually decreased from 2001. However, the number of publications on BBB disruption was still higher (2001–2010) than or equal to (2011–2020) the numbers of studies focusing on other strategies. Research on nanoparticles became a main research focus for improving BBB/BTB penetration in the past decade (2011–2020). Several techniques emerging from medical physics were applied in preclinical research on TCH (*n* = 10). With the improvement of microcatheters, superselective infusion techniques gradually became a routine treatment option in 2011–2020 (*n* = 14). In parallel, real-time image-based monitoring became increasingly important for optimization of therapeutic procedures (*n* = 14).

### Strategies for Improved Intra-Arterial Drug Delivery to Brain Tumors

#### BBB/BTB Disruption

The BBB represents a major obstacle for CNS drug delivery (32). The anatomy of BBB, mainly composed of brain endothelial cells, astrocytes, and pericytes (33), has been well studied. The tight junctions of endothelial cells are the mainstay of the barrier structure (34), while transcellular carriers and cell surface receptors (35) allow the selective transport of nutrients and metabolites (36) across the BBB. Morphology and function of BBB often change under pathological conditions (37). It has been found that the BBB is damaged in primary tumors such as meningiomas (38), schwannomas (39), and high-grade gliomas (40), but this does not necessarily facilitate drug uptake (41). On the other hand, excessive BBB permeability increases the risk of cerebral edema (42). Therefore, reasonable and targeted changes in BBB permeability are critical to increase drug uptake while at the same time minimizing drug dose and adverse effects.

Some chemical agents have been shown to modify the BBB (Table 1). The hypertonic solution mannitol (80) draws water from endothelial cells into blood vessels, causing endothelial cell shrink. This impacts tight junctions, allowing drugs to pass the BBB (81, 82). Reagents with comparable effects comprise arabinose (83) and hypertonic urea (84). To date, mannitol remains one of the most effective hyperosmotic solutions for transient BBB disruption. Mannitol is safe and well-tolerated in combination with intra-arterial chemotherapy (Table 2). The approach increases BBB permeability and thus facilitates the use of relatively large-sized antibodies (e.g., bevacizumab and cetuximab) for the treatment of malignant glioma (100, 101, 107). It improves therapeutic effects of methotrexate and carboplatin on primary CNS lymphomas and cerebral metastases (93, 98). The risk for cognitive impairment was reduced when mannitol-mediated BBB disruption was used together with radiotherapy in patients with germ cell tumors (113). A multi-center study also confirmed the benefits of intra-arterial chemotherapy with osmotic BBB disruption in enhancing the therapeutic response of primitive neuroectodermal tumors (94). By detecting ^99m^Tc-glucoheptonate (TcGH), SPECT scans confirmed BBB opening within 40 min after mannitol injection and returned to the steady state after 6–8 h. This provides a wide time window for clinical applications (82). However, preclinical studies revealed that BBB disruption with mannitol is variable (114) and may cause an unexpected increase in transcapillary transport of anticancer drugs into healthy brain tissues (45, 115). In both humans and experimental animals (e.g., pigs and rats), focal motor seizures occur during BBB opening and last for several hours after termination of the procedure. The presence of high-frequency and high-amplitude electroencephalography (EEG) signals suggests that intra-arterial injection of mannitol through the anterior circulation may directly affect the motor cortex, regardless of the size and location of the tumor, or applied chemotherapy (116, 117). Other potential complications such as tachycardia and increased intracranial pressure as well as nausea, headache, and vomiting have been reported retrospectively (118). BBB disruption may also result in transient aphasia, hemiparesis, or even edema-induced intracranial herniation (94). Some conditions can affect mannitol-mediated BBB opening (114). For example, low brain temperature and the Na^+^/Ca^2+^ exchange blocker KB-R7943 enhance mannitol-mediated BBB opening (119), while intraperitoneal administration of magnesium sulfate attenuates mannitol effects (120). Thus, hyperosmotic mannitol infusion remains challenging and is hard to control, even though most side effects seem mild and can be controlled by medications (98, 102).

**Table 1:** Agents for BBB modification: preclinical studies.

**No**.	**Agent**	**Concentration**	**Infusion velocity**	**Effect**	**Detection method**	**Model**	**References**
1	Mannitol	1.37 M, 1.60 M	2.50 ml over 30 s	Capillary permeability ↑, cerebral blood flow ↑, blood pressure ↑	^14^C-AIB, ^14^C-IAP	W256 carcinosarcoma and C6 glioma-bearing rats	(43)
		1.60 M	0.25 ml/s/kg for 30 s	Survival of the rats ↓	Methotrexate	Osteogenic sarcoma-bearing rats	(44)
		1.40 M	3.00 ml over 45 s	BBB opening ↑, leakage of HRP into tumors ↑, concentrations of EB, HRP in the normal brain ↑, concentrations of 5-FU in serum, tumor and tumor-free brain ↑	EB, HRP, 5-FU	RG-C6 glioma-bearing rats	(45)
		1.60 M	2.50 ml over 30 s	Concentrations, distribution in tumor and cortex ↑	^14^C-methotrexate	C6 glioma-bearing rats	(46)
		1.60 M	0.12 or 0.25 ml/s for 30 s	Tumor uptake of boron compounds ↑, mean survival time of the rats ↑	BSH or BPA, EB, HRP	F98 glioma-bearing rats	(47–52)
		1.60 M	0.09 ml/s for 30 s	Percentage of accessible tissue space ↑	^14^C-AIB, ^14^C-dextran 70, ^14^C-methotrexate	LX-l human small cell lung carcinoma-bearing female athymic nude rats	(53)
		not given	Not given	Survival time of the rats ↑	monoclonal antibody BR96-DOX	LX-l human small cell lung carcinoma-bearing female athymic nude rats	(54)
		1.60 M	1.5 g/kg	Perfusion territory and BBB disruption ↑	SAA (DHB, SA, DHTA)	Sprague–Dawley rats	(55)
2	Arabinose	1.60 M	0.78 ml over 30 s	BBB permeability ↑	EB	Male Fischer 344 rats	(56)
3	Bradykinin	10 mg/kg/min	53.3 μl/min for 15 min	Permeability in tumor capillaries ↑, blood volume in tumor or brain tissue =	^14^C-AIB, ^14^C-sucrose, ^14^C-inulin, ^14^C-dextran, EB, and HRP	9L gliosarcomas, C6 gliomas and RG-2 glioma-bearing rats	(57, 58)
4	RMP-7	0.1 mg/kg/min	53 μl/min for 15 min	Permeability in tumor capillaries ↑, blood volume in tumor or brain tissue =	^14^C-AIB, ^14^C-sucrose, ^14^C-inulin, and ^14^C-dextran	RG-2 glioma-bearing rats	(59)
		0.1 mg/kg/min	53 μl/min for 15 min	Permeability in tumor capillaries ↑↑, permeability of normal brain capillaries ↑, permeability of the vascular barriers to hydrophilic compounds ↑, lipophilic drug ↓	^14^C-carboplatin, ^14^C-dextran, and ^14^C-BCNU	Female Fischer 344 and Wistar rats	(60)
		0.1 mg/kg/min	53 μl/min for 15 min	Permeability in tumor capillaries ↑, survival of the rats ↑	^14^C-carboplatin, and ^14^C-dextran	RG-2 glioma-bearing rats	(61)
		0.1 mg/kg/min	53 μl/min for 15 min	Delivery of carboplatin to brain tumors↑, survival time of the rats ↑	^14^C-carboplatin	RG-2 glioma-bearing rats	(62)
		0.1 mg/kg/min	53 μl/min for 15 min	Cytokines delivery to brain tumors ↑	^125^I-IFN-gamma, TNF-alpha, and interleukin-2	RG-2 glioma-bearing rats	(63)
		1.5 mg/kg	For 15 min	Viral delivery to brain tumors ↑, normal brain tissue =	Herpes virus hrR3 encoding virus thymidine kinase gene and the lacZ reporter gene	9L gliosarcoma-bearing rats	(64)
5	Alkylglycerols	0.01–0.3 M	6 ml/min for 12 s	Methotrexate delivery to the brain and to brain tumors ↑	Cisplatin, methotrexate	C6 gliomas-bearing rats	(65)
6	1-O-pentylglycerol	300 mM	6 ml/min over 12–15 s	Concentrations of ErPC in the brain tumor ↑↑, brain tissue adjacent to tumors ↑	ErPC	C6 gliomas-bearing rats	(66)
		200 mM	6 ml/min for 2 min	Delivery of small and large compounds to normal brain and brain tumors ↑	Fluorescein sodium, RB 200-albumin, FITC-dextran, methotrexate	C6 gliomas-bearing rats	(67)
	1-O-pentylglycerol	10–300 mM	53 μl/min for 15 min	No signs of toxicity, delivery of methotrexate to the brain ↑	Methotrexate	Male Wistar rats	(68, 69)
	2-O-hexyldiglycerol	50–100 mM	53 μl/min for 15 min	No signs of toxicity, delivery of methotrexate to the brain ↑	Methotrexate	Male Wistar rats	(68, 69)
7	Histamine	1, 10 mg/kg/min	1.5 ml/h for 10 min	Regional tumor and brain capillary permeability ↑	^14^C-AIB	RG-2 glioma-bearing rats	(70)
8	Nifedipine	0, 0.1, 1, 5, 10 mg/kg/min	For 15 min	Permeability in tumor capillaries ↑	EB	Male Wistar rats	(71)
9	Etoposide	3.0–22.5 mg/kg	For 25 min	BBB permeability ↑	^99m^Tc-DTPA, EB	Sprague-Dawley rats	(72)
10	Cisplatin	1, 1.2, 1.5 mg	For 60 min	BBB permeability ↑, local cerebral blood flow =	^14^C-AIB, ^18^F- fluoroantipyrine	Female Wistar rats	(73)
11	Vinorelbine	5–10 mg/kg	4 ml/kg for 2 min	BBB permeability ↑	EB	Sprague-Dawley rats	(74)
12	Leukotriene C4	2.50%	53.3 μl/min for 15 min	BBB/BTB permeability ↑	^14^C-AIB, γ-GTP	RG-2 glioma-bearing rats	(75)
13	Leukotriene E4	5 μg/ml	53.3 μl/min for 15 min	Permeability in tumor capillaries ↑, permeability of normal brain capillaries =	^14^C-AIB, ^14^C-sucrose, ^14^C-5-FU, and ^3^H-methotrexate	C6 glioma-bearing rats	(76)
14	TNF-alpha	0, 1,000, 10,000, 100,000 IU	for 1, 2, 4, 8, and 16 h	BBB permeability ↑	Sodium fluorescein, EB-albumin	newborn pigs	(77)
15	Short-acting NO donor (Proli/NO)	10^−2^-10^−12^ M	For 30 s and 3 min	BBB permeability ↑, long-term survival of the rats ↑	^14^C-AIB, ^14^C-sucrose, ^14^C-dextran, sodium nitrite, carboplatin	C6 glioma-bearing rats	(78)
16	Papaverine	0.5 mg/kg	For 0.5, 1, 2, 3 and 5 h	BBB permeability ↑	Occludin, claudin-5, F-actin, PKA, HSP70	C6 glioma-bearing rats	(79)

*AIB, aminoisobutyric acid; BBB, blood-brain barrier; BCNU, carmustine; BPA, boronophenylalanine; BSH, sodium borocaptate; BTB, blood-tumor barrier; DHB, 2,4-dihydroxybenzoic acid; DHTA, 2,5-dihydroxyterephthalic acid; DTPA, Diethylene triamine pentaacetic acid; DOX, doxorubicin; EB, Evans blue; ErPC, erucylphosphocholine; FITC, fluorescein isothiocyanate; HRP, horseradish peroxidase; HSP70, heat shock protein 70; IAP, iodoantipyrine; IFN, interferon; PKA, protein kinase A; RB 200, rhodamine B200; γ-GTP, gamma glutamyl transpeptidase; SA, sodium salicylate; SAA, salicylic acid analogs; TNF, tumor necrosis factor; 5-FU, 5-fluorouracil*.

**Table 2:** Agents for BBB modification: clinical research and applications.

**No**.	**Agent**	**Tumor**	**Intra-arterial administration**	**Surgery**	**Radiation**	**Systemic chemotherapy**	**Analysis method**	**Effect**	**References**
1	Mannitol (1 ml/s, 120 ml)	Glioblastoma	5-fluarouracil, Adriamycin	×	×	×	CT	Tumor mass size ↓/=	(85)
	Mannitol (8–10 ml/s, 30 s)	Glioblastoma	Methotrexate	×	×	×	Cox Proportional Hazards Regression Model	Survival of the patients ↑	(86)
	Mannitol (25%, 30 s)	Malignant gliomas, primary CNS lymphoma	Methotrexate	×	×	×	CT and ^99m^Tc- SPECT	Tumor area↓, BBB/BTB disruption ↑	(87)
	Mannitol (20%, 200 ml)	Brain metastasis	ACNU				Two- and one-compartment open model, high-performance liquid chromatography	ACNU levels in blood and tissue ↑	(88)
	Mannitol (8–10 ml/s, 30 s)	Brain metastasis	Methotrexate			×	CT and ^99m^Tc-glucoheptonate radionuclide scans	BBB/BTB disruption ↑	(89)
	Mannitol (20%, 200 ml)	Astrocytoma	ACNU	×	×		CT and high-performance liquid ion exchange chromatography	2/3-year survival rate of the patients ↑, ACNU levels in blood and tissue ↑	(90)
	Mannitol (25%, 27–30 s)	Astrocytoma, glioblastoma	Methotrexate			×	Three-compartment model, fluorescence polarization immunoassay	Methotrexate concentrations in serum and urine ↑, methotrexate half-life and cytotoxic concentrations ↑	(91)
	Mannitol (20%, 50 ml)	Malignant gliomas, brain metastases	ACNU and cisplatin		×		CT	Survival of the patients with malignant gliomas =, with brain metastases ↑	(92)
	Mannitol (25%, 4–10 ml/s, 30 s)	Primary CNS lymphoma, primitive neuroectodermal tumor, metastatic disease, germ cell tumor, glioblastoma multiforme	Carboplatin or methotrexate			×	MRI, CT, Karnofsky performance status evaluation	Tumor volume ↓, median survival times ↑	(93–95)
	Mannitol (25%, 3–11 ml/s, 30 s)	Primary CNS lymphoma, primitive neuroectodermal tumor	Carboplatin or methotrexate			×	CT, ^99m^Tc- glucoheptonate -SPECT	Time course to closure of the BBB disruption	(82)
	mannitol (25%, 3–12 ml/s, 30 s)	Primitive neuroectodermal tumors, medulloblastomas, germ cell tumors	Carboplatin or methotrexate		×	×	Physical examinations, CT, and/or MRI scans, cerebrospinal fluid studies, and ophthalmologic evaluations	Overall survival ↑, time to progression ↑, and neurocognitive function ↑ of the patients	(96)
	Mannitol (25%, 3–12 ml/s, 30 s)	Refractory anaplastic oligodendroglioma and oligoastrocytoma tumors	Carboplatin and melphalan			×	CT, MRI, audiologic, ophthalmologic and neuropsychologic evaluations, tumor response, duration of response, and survival	Adverse events ↓, tumor response ↑. median overall/progression-free survival of the patients ↑	(97)
	Mannitol (1,400 mg/m^2^, 10 min or 25%, 30 s)	Primary CNS lymphoma	Methotrexate			×	CT, fluorescence polarization immunoassay, non-compartmental analysis	Cerebrospinal fluid/serum ratio of methotrexate ↑, overall response rate ↑, median overall survival of the patients ↑, median progression-free survival of the patients ↑	(98, 99)
	Mannitol (25%, 4–6 ml/s, 30 s)	Primary CNS lymphoma	Carboplatin			×	DC-EEG, near-infrared spectroscopy	Lateralized DC-EEG response from negative to positive shift ↑, BBB disruption ↑	(16)
	Mannitol (20%, 60–90 ml)	Glioblastoma multiforme, astrocytoma, metastatic brain tumor	Fluorescein				Cerebral fluorescein micro-angiograms	Transport of fluorescein into tumors and normal brain tissue ↑	(47)
	Mannitol (25%, 5, 10 ml/120 s)	Recurrent or progressive malignant gliomas, malignant brainstem gliomas	Bevacizumab (superselective)				MRI	Tumor area, volume, perfusion ↓, progression-free survival of the patients ↑	(100–104)
	Mannitol (25%, 10 ml/120 s)	Recurrent glioblastoma multiforme	Bevacizumab, cetuximab, temozolomide (superselective)	×	×	×	MRI, PET	Tumor size and ^18^F-FDG uptake ↓	(105)
	Mannitol (25%, 10 ml)	Multiply recurrent pediatric ependymoma	Bevacizumab, cetuximab (superselective)	×	×	×	MRI, PET	Residual tumor size and activity ↓	(106)
	Mannitol (20%, 12.5 ml/120 s)	Recurrent malignant gliomas	Bevacizumab (superselective)		×	×	MRI	Tumor progression ↓	(14)
	Mannitol (20%, 12.5 ml/120 s)	Recurrent malignant gliomas	Cetuximab (superselective)				Physical and neurological examination, MRI	Safe and tolerated toxicity	(107)
	Mannitol (20%, 12.5 ml/120 s)	Newly diagnosed glioblastoma	Cetuximab (superselective)	×	×	×	MRI	No evidence of tumor progression or recurrence, less complications (e.g., mild headache)	(108)
2	RMP-7 (0.1, 0.3, 1, 3 mg/ml, 1 ml/min)	Recurrent malignant gliomas	None		×		^68^Ga EDTA-PET, MRI	Transport of ^68^Ga EDTA into tumors ↑, normal brain tissue -tumor volume ↓	(109)
	RMP-7 (10, 30, 100, 300 ng/kg)	Recurrent malignant gliomas	Carboplatin				Gadolinium-enhanced MRI	Tumor volume ↓	(110)
	RMP-7 (300 ng/kg)	Recurrent glioblastoma multiforme, anaplastic astrocytoma	Carboplatin	×			MRI	Tumor progression and survival differ in patients with hypervascular and hypovascular tumors	(111)
	RMP-7 (11,448 ng/m^2^)	Glioblastoma multiforme, adenocarcinoma, high-grade astrocytoma, mixed anaplastic glioma	Carboplatin	×	×	×	MRI	No obvious tumor mass ↓, safe and tolerated neurological complications	(112)

*ACNU, nimustine; BBB, blood-brain barrier; BTB, blood-tumor barrier; CNS, central nervous system; DC-EEG, direct-current electroencephalography; EDTA, ethylenediamine tetraacetic acid; FDG, fluorodeoxyglucose*.

The BTB is found on tumor capillaries and can have a continuous non-fenestrated, continuous fenestrated or discontinuous phenotype (121). The surface receptor profile of BTB capillaries is more heterogeneous than that of normal capillaries (41, 122). Intra-arterial infusion of bradykinin (57), its analog RMP-7 (known as Cereport^®^ or Lobradimil, a widely used adjuvant chemotherapy reagent) (59), 1-O-pentylglycerol (66), or calcium antagonists (71) into rats transplanted with different tumor cell lines significantly increases BTB permeability. However, blood volumes within the tumor or the surrounding brain remain unchanged (Table 1). RMP-7 penetrates the BTB by activating B2 receptors on endothelial cells (60), which seems to be regulated by the NO-cyclic GMP pathway (123, 124). Infusion of RMP-7 also enhances intra-arterial delivery of a therapeutic herpes simplex virus vector (64, 125) and tumor uptake of carboplatin (61, 126). Short-chain alkylglycerols promote delivery of methotrexate (68) (Table 2). In Fischer 344 rats with gliosarcoma, bradykinin fosters selective herpes simplex virus infection in multiple tumor foci and increases the absorption of single crystal iron oxide nanoparticles (127). Moreover, histamine has a selective effect on increasing BTB permeability that is mediated by H2 receptors (70). Animal experiments demonstrated that intra-carotid infusion of leukotriene C4 (75) and E4 (76) increase BTB permeability, but do not affect normal brain capillaries. Preclinical evaluation of RMP-7 indicated a high efficacy in tumor uptake but minimal disturbance to normal tissues. It can, however, cause rapidly emerging side effects including hypotension, hypertension, abdominal pain, vasodilatation, headache, nausea, tachycardia, fatigue, and vomiting during intravenous infusion in a dose-dependent manner (128). RMP-7 may also cause leukopenia, nausea, thrush, cellulitis, urinary tract infection, hematuria, weakness, seizures, sensory loss, cortical blindness, oculomotor nerve palsy, and even ischemic stroke at the maximum intra-arterial infusion dose of 300 ng/kg (110, 112). However, the RMP-7-induced BBB/BTB permeability is transient and the barrier can spontaneously recover even during RMP-7 administration. This makes it difficult to define the optimal dosing and timing paradigm of RMP-7 in order to promote the maximum intratumoral concentration of chemotherapeutic agents (129, 130). Therefore, any potential beneficial effects from RMP-7-mediated drug delivery need to be confirmed in further clinical investigations.

Importantly, drugs used for tumor treatment may also exert effects on the BBB (Table 1). Intra-carotid infusion of vinorelbine into rats increases local BBB permeability at high doses (74), although recent data show much lower vinorelbine efficacy in brain vs. peripheral metastases (131). Cisplatin (73) and etoposide (72) were reported to open the BBB, and this effect precedes changes in local cerebral blood flow and necrosis. BBB opening may therefore be a sensitive indicator of cisplatin and etoposide neurotoxicity during intra-arterial administration (73). However, cisplatin is known for its acute gastrointestinal toxicity causing nausea and vomiting and can lead to myelosuppression (132). Etoposide has systemic side effects, including leukopenia, thrombocytopenia, and anemia when used with carboplatin (133). Another main obstacle that restricts further application of cisplatin and etoposide is their unknown mechanism. Whether the capability of these drugs to bypass the BBB is due to increased bradykinin release or P-glycoprotein expression in newly formed vessels (134) remains uncertain and requires future research.

Recently, it has been found that convection-enhanced delivery (CED) using catheters stereotactically inserted into brain tumors fosters drug delivery into these tumors and surrounding brain tissue by establishing a local positive pressure gradient (135). In contrast to diffusion-based concentration gradients, CED has advantages when applying compounds of high molecular weight because of a BBB bypass (135, 136). A main application of CED is the targeted delivery of cytotoxin to the tumor parenchyma or the surrounding tissues with encouraging results being reported for glioblastoma multiforme (137, 138). However, there is not yet convincing evidence for a benefit of CED in other chemotherapeutic paradigms even though preliminary experience is promising. In a F98 glioma rat model, CED of platinum-based drugs and liposomes increases drug accumulation in tumor tissue and extends the median survival time (139). CED of carboplatin has also been reported to have a sound therapeutic effect on glioblastomas in a Phase I clinical trial (140). However, there are a number of side effects caused by CED, such as headache, seizure, fever, nausea, vomiting, fatigue, erythema, and even liver enzyme perturbations and hematological changes (141, 142), which are related to the time and location of the treatment (143). Factors such as catheter reflux, leakage, or improper imaging guidance may lead to treatment failure (144).

Focused ultrasound is a novel non-invasive strategy for reversibly disrupting BBB tight junctions. Application of low frequency continuous wave ultrasound (~10 ms bursts, 1 MPa amplitude, 1 Hz frequency for 20–30 s intervals) generated well-controllable damage to tumor tissue, but minimal disturbance to surrounding healthy brain tissue (145). Microbubbles are standard ultrasound contrast agents and can help to create large shear microstreaming to open the capillary wall (146). Doxorubicin encapsulated in liposomes significantly accumulates in the rat brain after MRI-guided focused ultrasound with microbubbles (147) and has been investigated in a patient with high-grade glioma recently (148). Using the same strategy, focused ultrasound enhances the delivery of BCNU by intravenous administration to normal and C6 glioma-implanted rat brain tissue (149) as well as methotrexate to the rabbit brain (150). The most severe side effects are necrosis, febrile seizures, hemorrhage, and brain edema. Other complications include tolerable back pain and self-limiting headache (151, 152). Focused ultrasound may offer a promising method greatly improving local drug delivery after intra-arterial administration, but more evidence for clinical safety, efficacy, and feasibility is required.

The recent advent of nanotechnology has led to widespread nanoparticle applications in intra-arterial administration (Table 3). Due to the ability to pass through the BBB, some nanoparticles can be used for intra-arterial drug delivery (168). The cationic (positive) charge on their surface increases homing toward negatively charged tumor cell surfaces (169). It has been demonstrated that intra-arterial injection of paclitaxel-loaded cationic, polymeric, and magnetic liposomes increases drug delivery to the tumor tissue (156). When combined with BBB disruption induced by focused ultrasound, intra-arterial liposome injection increases their deposition into tumor tissue of C6 tumor-bearing rats (158). Similarly, application of cationizable lipid micelles (163) with cationic short peptides such as the cell-penetrating trans-activator of transcription (TAT) was shown to increase the uptake of micelles (165) and can be used for selective drug delivery to gliomas. The translocation efficiency of nanoparticles is not only determined by surface charge. For instance, larger particles are more likely to adhere to the vessel wall, but the hydrodynamic resistance for BBB crossing is also increased (162). Other parameters such as shape, lipophilicity, and ligand density can affect effective BBB penetration of nanoparticles (170). More research is needed to optimize intra-arterial nanoparticles delivery protocols for future applications.

**Table 3:** Microparticles for BBB penetration: preclinical studies.

**No**.	**Microparticle**	**Combined technique**	**Detection method**	**Effects**	**Model**	**References**
1	Cationic magnetic aminodextran microspheres and neutral dextran microspheres	Magnetic field of 0.6 Tesla	Fe_3_O_4_ magnetite atomic absorption	Magnetite concentrations in tumor ↑, and in non-target tissue ↓	RG-2 glioma-bearing rats	(153)
2	BCNU-loaded wafer and PLA nanoparticles coated with transferrin	None	Transferrin X-ray photoelectron spectroscopy, Bratton-Marshall colorimetric assay and zeta-potential analysis, ^99m^Tc-SPECT, Gd-DTPA-MRI	Tumor growth ↓, average survival time of the rats ↑	C6 glioma-bearing rats	(154)
3	(DSPE-mPEG2000-) ferrociphenol-loaded lipid nanocapsules	CED	MRI/MRS	Survival time of the rats ↑, accumulation of lipid nanocapsules in the tumor zone ↑	9L gliosarcoma-bearing rats	(155)
4	Paclitaxel-loaded cationic polymeric magnetic liposomes	Magnetic field of 0.5 Tesla	High-performance liquid chromatography	Brain concentration of the liposomes ↑	Sprague-Dawley rats	(156)
5	Cisplatin, oxaliplatin-loaded liposomes	BBB opening, gamma knife irradiation	MRI, inductively coupled plasma mass spectrometry	Accumulate of the liposomes in brain tumors ↑, mean survival time of the rats ↑, tumor control ↑	F98 glioblastoma-bearing rats	(157)
6	Cisplatin-loaded liposomes	CED, gamma knife irradiation	Inductively coupled plasma mass spectrometry	Maximum tolerated dose ↑, median survival time of the rats ↑ accumulation of drugs in tumor ↑, systemic toxicity ↓	F98 glioblastoma-bearing rats	(139)
7	Doxorubicin-loaded liposomes	Focused ultrasound with microbubbles	MRI, fluorometric assay	Tissue concentration of doxorubicin ↑	Sprague-Dawley rats	(147)
8	Anionic, cationic, and charge-neutral liposomes	Focused ultrasound, TCH	Diffuse reflectance spectroscopy, multispectral imaging, spatial frequency-domain imaging	Uptake of the liposomes by brain tumor ↑	C6 glioma-bearing rats	(158–161)
9	Large (200 nm) and small (60–80 nm) fluorescent dye-loaded liposomes	TCH	Diffuse reflectance spectroscopy, multispectral fluorescence imaging	Uptake of the liposomes by brain tumor ↑	C6 glioma-bearing rats	(162)
10	Cationizable micelles	TCH	Diffuse reflectance spectroscopy	Uptake of the micelles by brain tumor ↑	9L gliosarcoma-bearing rats	(163)
11	TAT	TCH	Multispectral fluorescence imaging	Uptake of the TAT by brain tumor ↑	9L gliosarcoma-bearing rats	(164)
12	TAT-decorated and neutral micelles	FA	Diffuse reflectance spectroscopy	Deposition of both micelles in the tumor and blood vessels ↑	9L gliosarcoma-bearing rats	(165)
13	Magnetically-mediated retention of iron oxide nanoparticles	Magnetic field of 0.15 or 0.35 Tesla	MRI, electron spin resonance spectroscopy	Nanoparticle accumulation in the tumor ↑	9L gliosarcoma-bearing rats	(166)
14	Heparin-coated superparamagnetic nanoparticles loading cationized model protein β-galactosidase	Magnetic field of 0.35 Tesla	MRI, β-galactosidase activity spectrophotometry	Nanoparticle accumulation in the tumor ↑, exposure of normal brain regions ↓	9L gliosarcoma-bearing rats	(167)

*CED, convection enhanced delivery; DSPE-mPEG2000, 1,2-Distearoyl-sn-glycero-3-phospho-ethanolamine-N-[methoxy-(polyethylene glycol)-2000]; FA, flow arrest; Gd-DTPA, gadolium-diethylenetriamine pentaacetic acid; PLA, poly(D,L-lactic acid); TAT, cell-penetrating trans-activator of transcription; TCH, transient cerebral hypoperfusion*.

#### TCH

The pharmacokinetic theory of intra-arterial drug administration suggests that a comparable tumor response can be obtained faster by intra-arterial than by intravenous administration due to the first pass effect. Regional slow blood flow can increase tissue drug levels and rapid systemic clearance (171). In a non-human primate model, phased pulsatile infusion during the diastole resulted in excellent distribution of the drug in blood (172). Intra-arterial drug administration during cerebral hypoperfusion promotes drug deposition in tumor and surrounding tissues, increases the contact time of drugs with tumor cells, and avoids non-targeted binding to plasma proteins (173). Hence, such improved methods increase drug concentrations in the tumor tissue while at the same time enabling reduction of the overall amount of drugs infused, mitigating potential side effects (174, 175).

Almost 30 years ago, it was shown that glucose reduces the pH of tumors by reducing blood flow, which in turn promotes thermochemotherapeutic effects (176). Various vasodilators such as adenosine (177), histamine (178), or iloprost (179) have been considered to alter regional cerebral blood flow in brain and tumor capillaries. Adenosine was shown to promote endovascular embolization during arteriovenous malformation by transient induction of hypotension (180). Taking advantage of this, a decrease in mean arterial blood pressure can be achieved by intravenous injection of adenosine, esmolol, and large doses of cold saline. When combined with bilateral carotid occlusion, cerebral blood flow measured by a laser Doppler probe would transiently drop to 10–20% for 30–40 s and completely recover within 5 min without inotropic support. This procedure allows intra-arterial injection of less mannitol to achieve BBB disruption lasting at least 60 min (174). It has been shown that TCH can improve the uptake of lipophilic drugs (e.g., BCNU) into rabbit brains without causing severe neuronal damage or abnormal EEG signals (181). In glioma-bearing rats, it was found that a combination of lowering cerebral blood flow and BBB opening increases the concentration of the chemotherapeutic drug (e.g., mitoxantrone) in glioma tissue more than 10-fold as compared to the contralateral, tumor-free brain tissue. The treatment effect was reported to last for more than 4 h (175). Furthermore, TCH is sufficient to enhance early regional deposition of nanoparticles such as micelles (163–165) and liposomes (158–162) into tumor tissues. This provides a novel approach for targeted intra-arterial tumor therapy (Table 3).

Despite these promising findings, the translation of TCH-promoted intra-arterial drug administration to brain tumors into clinically applicable procedures is still not completed. Although the strategy has been validated in standardized animal models, tumor response and survival time after intra-arterial drug administration in humans were not yet described. TCH has been widely used in neurosurgical procedures for the treatment of arteriovenous malformations and aneurysms, but drug distribution in tumor vessels is complex and blood flow might be variable. Moreover, a relatively high risk of systemic hypotension or regional low cerebral blood flow is a challenge for the clinical application of TCH in intra-arterial drug administration.

#### SIACI

In theory, primary brain tumors usually have a limited number of supplying arteries. This is advantageous when considering treatment by superselective intra-arterial infusion (182). However, since the ophthalmic artery originates from the internal carotid artery, drugs injected into the internal carotid artery also reach the eye, which can injure retinal ganglion cells and lead to temporary or permanent ocular complications (183) such as vision loss, vasculitis, and cataracts (184). Hence, it is reasonable to selectively inject into the main tumor-supplying arteries rather than infusing into major brain supplying vessels, such as the carotid or vertebral arteries (185). In clinical practice, the tip of a superselective catheter is usually placed in the A1 segment of the anterior cerebral artery, the M1 segment of the middle cerebral artery, or in the posterior communicating artery (182). Supraophthalmic carotid infusion became the preferred procedure to prevent drugs from entering the eye (184, 186–191). Flow-directed soft-tipped balloon or single-lumen catheters (188) with an extended tip (182–184) can be remotely controlled by hydraulic forces (189) and allow to maneuver even in extremely curved skull base vessels (184). It has been reported that supraophthalmic carotid chemotherapy can be used to treat malignant gliomas with low dose cisplatin and BCNU (182, 191, 192), and that the pharmacokinetic changes of ^11^C-BCNU are consistent with the metabolic changes captured by PET (185). In combination with external beam radiation therapy, supraophthalmic infusion of fluorouracil was reported to achieve acceptable median survival rates in anaplastic astrocytomas and glioblastomas (193). Superselective intra-arterial infusion of bevacizumab (100, 103) or cetuximab (107, 108) combined with mannitol-mediated BBB opening exerts profound anti-proliferative effects (194) and reduces tumor volume. The procedure was proven to be safe and effective in the treatment of brainstem gliomas (104) and multiply recurrent pediatric ependymoma (106) as well as vestibular schwannomas (195). However, it is still unclear whether long-term slow infusion or rapid bolus infusion is the more effective approach (182). It was recently shown that median progression-free survival of patients with recurrent glioblastoma receiving a single dose of SIACI 15 mg/kg bevacizumab and mannitol is comparable or even longer than that of those who received biweekly intravenous bevacizumab at 10 mg/kg (102). Moreover, side effects including epilepsy and headache indicate a need for careful dose adjustments in therapies relying on multiple administrations (102). Adverse events during invasive procedures further include nausea, bradycardia, vomiting, headache, and vascular complications such as asymptomatic subintimal tear, and even intracranial herniation, stroke, and cerebral hemorrhage. Thus, extensive experience in endovascular intervention is required to apply superselective intra-arterial drug administration (94, 133). Favorable results have been reported for a patient suffering from metastatic breast cancer. Skull and dura mater metastasis disappeared after repeatedly performed superselective intra-arterial administration of epirubicin into the right superficial temporal artery and the middle meningeal artery, combined with embolization of tumor-supplying vessels (196). Moreover, the median overall survival was effectively extended to 6 months in small-cell lung cancer patients with brain metastases receiving superselective intracranial arterial infusion chemotherapy (teniposide, ACNU, and carboplatin) (197) vs. 4–6 months of standard whole brain radiotherapy (198, 199). Therefore, carefully weighing beneficial outcomes vs. potential risks and adverse events is required for different brain tumor types. Additional comparison to system administration in further clinical trials is also warranted.

#### Advanced Imaging Technology

Advanced imaging technology is of great value for the diagnosis and treatment of brain tumors (Table 4). Originally, imaging technology was mainly used to detect the radioactivity of radiopharmaceuticals or contrast agents in order to reflect the distribution of drugs in tumor tissues. Later, imaging was used to guide the intra-arterial drug perfusion and to predict therapeutic effects. Dynamic PET imaging was first performed to determine the position of the superselective catheter by intra-arterial injection of ^11^C-labeled BCNU, and to predict clinical response by comparing ^11^C radioactivity within the tumor after SIACI vs. intravenous infusion (185). Similarly, continuous PET scans were performed to quantify the pharmacokinetic advantage of ^13^N-labeled cisplatin by calculating the time course of ^13^N activity in tumor and normal brain tissue (200). In addition, PET can distinguish between cerebral necrosis following supraophthalmic intra-arterial chemotherapy or radiotherapy (low metabolic turnover) and tumor recurrence (high metabolic turnover) using ^18^F-fluorodeoxyglucose (^18^F-FDG) (201). Glucose metabolism was also measured by PET imaging to evaluate early treatment effects of intra-arterial administration of ACNU into glioblastomas and astrocytomas (202), as well as recombinant human tumor necrosis factor (TNF)-alpha in malignant astrocytomas (203). Another non-invasive method using radiopharmaceutical labeling to monitor drug biodistribution in tumors for assessing the effectiveness of intra-arterial chemotherapy is dynamic scintigraphic imaging with ^195m^Pt-labeled cisplatin (206). Moreover, SPECT using ^99m^Tc-hexamethyl-propyleneamine oxime (HMPAO) allows one to evaluate the cerebral distribution after either fast pulsatile or slow continuous supraophthalmic carotid infusion (186). Using ^99m^Tc-hexakis-2-methoxyisobutyl-isonitrile (MIBI), SPECT can also assess changes in tumor MIBI uptake before and after radiochemotherapy (204).

**Table 4:** Imaging techniques for intra-arterial drug delivery.

**No**.	**Imaging modality**	**Tumor types**	**Intra-arterial administration**	**Results(s) and effect(s)**	**References**
1	PET	Malignant gliomas of grade III or IV	^11^C-BCNU (superselective)	Half-lives for the second curve components ↓, chemical and metabolic decomposition ↑ of BCNU	(185)
		Glioblastoma	^13^N-cisplatin	Ratio of integrated tumor/brain count ratios ↑	(200)
		Glioblastoma multiforme, astrocytoma, oligodendroglioma, diffuse CNS leukemia, and metastases	BCNU, ^18^F-FDG	Glucose metabolic values of the necrotic areas ↓	(201)
		Glioblastoma and astrocytoma	ACNU, ^18^F-FDG	Regional cerebral metabolic rate for glucose ↓	(202)
		Malignant astrocytoma	Recombinant human TNF-alpha, hemoglobin-bound C^15^O (for blood volume), ^18^F-FDG (for glucose metabolism)	Cerebral hemocirculation and glucose metabolism in tumor ↓	(203)
2	SPECT	Recurrent malignant gliomas	ACNU or cisplatin and etoposide, ^99m^Tc-HMPAO	Homogeneous distribution with fast pulsatile infusion, inhomogeneous distribution with slow continuous infusion	(186)
		Malignant astrocytoma, meningioma, lymphoma, germ cell tumor, metastasis, schwannoma, ependymoma	^99m^Tc-MIBI	MIBI-index immediately following therapy correlated with treatment response 2 months after therapy	(204)
	SPECT, MRI	Cystic anaplastic astrocytoma, glioblastoma multiforme	ACNU and cisplatin, ^99m^Tc-HMPAO	Concentrations in the tumor ↑	(205)
3	dynamic scintigraphic imaging	Recurrent pineal blastoma, progressive oligodendroglioma, and recurrent high-grade astrocytoma (grade III or IV)	^195m^Pt-cisplatin	Higher local concentration of free cisplatin in tumors ↑	(206)
4	DC-EEG	Primary CNS lymphoma	Carboplatin	Lateralized DC-EEG response from negative to positive shift to monitor blood–brain barrier disruption	(16)
5	angio-CT	Astrocytoma	ACNU, iopamidol	Contrast enhancement of the tumor ↑, the medullary veins of the tumors ↑	(207)
		Brain metastases from hepatocellular carcinomas	Cisplatin, iopamiron	Tumor perfusion in the vascular territory ↑	(208)
6	phosphorus MRS	Recurrent mixed astrocytoma/oligodendroglioma	BCNU (superselective)	Phosphocreatine and phosphodiesters ↓, intracellular pH ↑	(209, 210)
	MRI, ^1^H-MRS	Recurrent glioblastoma	Bevacizumab (superselective)	Metabolic tCho/NAA ratio in the tumor ↓	(194)
	2D thick-slice MR-DSA	Meningiomas, gliomas, metastatic tumors, neuromas, and hemangioblastomas	Gadolinium chelates	Visualization of large cerebral arteries, venous sinuses, and most tributaries ↑, display of location, shape, and phase of tumors ↑	(211)
	MRI, PET	Recurrent butterfly glioblastoma	Bevacizumab (superselective)	Direct visualization of the brain parenchyma perfused for drug targeting and brain accumulation	(212)
	MRI	Retinoblastoma	Melphalan, topotecan and carboplatin	To monitor tumor size and the risk factors of abnormal enhancement of the postlaminar optic nerve	(213)
	MRI	Brain metastases from MDA-MB-231 breast tumor in the mouse model	Docetaxel	Mean tumor apparent diffusion coefficient values ↑, tumor volume ↓	(214)
	MRI	C6 glioma in the rat model	MSCs expressing ferritin heavy chain and enhanced GFP	To track the tropism and fate of MSCs after transplantation	(215)
	MRI, electron spin resonance spectroscopy	9L gliosarcoma in the rat model	Magnetically-mediated retention of iron oxide nanoparticles	Nanoparticle accumulation in the tumor ↑	(166)
	MRI, ultrasonic photoacoustic imaging	U87 glioma in the rat model	MSCs (conjugated with GFP) labeled with superparamagnetic iron oxide nanoparticles coated with gold	MSCs tracking after transplantation possible, tumor size ↓	(216)

*ACNU, nimustine; BCNU, carmustine; CNS, central nervous system; DC-EEG, direct-current electroencephalography; FDG, fluorodeoxyglucose; GFP, green fluorescent protein; HMPAO, hexamethyl-propyleneamine oxime; MIBI, hexakis-2-methoxyisobutyl-isonitrile; MSCs, mesenchymal stem cells; NAA, α-naphtalene acetic acid; tCho, total cholesterol; TNF, tumor necrosis factor*.

The main limitation of SPECT and PET is the relatively low spatial resolution of these imaging modalities. CT is superior in this respect, has widespread availability, and is easier to apply. The permeability of blood vessels in brain tumors can be assessed in CT images by measuring iopamidol distribution in blood vessels and the extracellular fluid space (217). Angio-CT provides more accurate information on the vascular territory of the tumor than digital subtraction angiography (DSA), which aids intra-arterial chemotherapy of metastatic brain tumors (208). Superselective angio-CT was also shown to precisely define enlarged medullary veins in patients suffering from astrocytomas (207). Recently, direct-current (DC)-EEG was applied for real-time, non-invasive monitoring of BBB opening during clinical treatment of a primary CNS lymphoma by measuring low frequency oscillations of 0.01–0.15 Hz (16), but the accuracy of the procedure needs to be verified.

With the widespread availability of MRI, the intra-arterial administration of therapeutic drugs to brain tumors is further improved. Due to its high resolution and sensitivity, MRI has the ability to accurately assess the therapeutic response to the tumors. Magnetic resonance spectroscopy (MRS) is used to reveal intracellular pH changes after BCNU treatment for anaplastic astrocytomas or glioblastomas with alkalization in intra-arterial administration and acidification in intravenous chemotherapy (209). The reduction of phosphocreatine and phosphodiesters, indicating early metabolic changes after intra-arterial treatment, can be visualized by MRS imaging and precedes apparent structural changes (210). Recently, proton MRS was successfully applied to detect a decreased total cholesterol (tCho)/α-naphtalene acetic acid (NAA) metabolite ratio. tCho is used as a marker for cell membrane breakdown and proliferation, while NAA indicates neuronal density and viability. The decreased tCho/NAA ratio was observed in two regions of interest (enhancing component and non-enhancing T2-hyperintense signal abnormality) after SIACI of bevacizumab in the treatment of recurrent grade IV glioblastoma (194). By measuring biochemical changes rather than detecting anatomical abnormalities, MRS avoids the non-specific reduction of MRI contrast enhancement by bevacizumab, while providing potential biomarkers of treatment efficacy (194). However, these preliminary studies lack a precise description of clinically meaningful endpoints, which are urgently needed for the long-term follow-up of larger patient populations to determine the correlation of early metabolic changes with treatment outcomes.

MRI usually determines the changes in tumor volume by measuring the size of the contrast-enhanced lesion. However, given that both recurrent tumors and therapeutic drugs may cause BBB destruction leading to heterogeneous enhancement and T2/FLAIR hyperintensity, MRI has limitations in distinguishing between tumor recurrence and long-lasting sequelae of the therapeutic intervention. To overcome this deficiency, hemodynamic changes in tumors are considered an important indicator to accurately predict disease progression. Originally, MR DSA following bolus injection of gadolinium chelates offers a high temporal resolution for showing large cerebral arteries, venous sinuses, and tumor blood vessels. In some doubtful cases, MR DSA was able to identify meningioma and acoustic neuroma by first-pass stain. The tumors were later confirmed by pathological examination (211). Next, dynamic susceptibility contrast MR imaging (DSC-MRI) became a more common MRI perfusion technique. It can reveal cerebral blood volume, cerebral blood flow, and mean transit time for assessing brain tumors by using gadolinium-based tracer kinetic and dilution models (218). MRI with gadolinium was recently used to provide a better visualization of ambiguous tumor feeding arteries during microcatheter injection of bevacizumab in treating a recurrent butterfly glioblastoma, underlining the role of MRI in real-time guidance of neurointervention (212).

However, use of gadolinium-based agents is associated with a potential risk for brain deposition and nephrogenic systemic fibrosis (219). Chemical exchange saturation transfer (CEST), a new contrasting strategy, can apply a saturation pulse to the resonance frequency of the exchange protons on the compound to sensitively detect perfusion area and BBB opening. Salicylic acid analogs (SAA) are the natural contrast agents used for CEST MRI and may offer increased safety and operability than gadolinium-based agents (55). In recent years, 3D pseudo-continuous arterial spin labeling (3D-pcASL), a non-invasive MR perfusion measurement technique not requiring contrast injection, has been found to provide higher image quality with less susceptibility artifacts than DSC-MRI. Cerebral blood volume and flow measured by DSC-MRI or 3D-pcASL are different between patients with tumor recurrence and those who experience long-lasting treatment effects (220). However, image distortion still occurs in inhomogeneous regions. To address this, the combination of 3D-pcASL and Turbo Spin Echo using Cartesian acquisition with spiral profile reordering was used to provide better contrast between tumor and normal tissues. Another benefit is high reproducibility between scanning sessions, suggesting a potential use for therapeutic response assessment (221).

MRI monitoring and magnetic targeting also play an important role for intra-arterial drug delivery systems. A prominent example is highly selective deposition of magnetic microparticles in brain tumors (Table 3). In the late 1980s, large multivesicular liposomes containing magnetic resonance contrast agents were found to spontaneously attach to the vascular bed of vessels in the frontal and occipital lobe when injected intra-arterially into experimental animals (222). Currently, intravenously infused iron oxide nanoparticles can be selectively deposited in gliosarcomas when using a low-strength magnetic field (0.4 T) (223). The short plasma half-lives of magnetic nanoparticles make them more suitable for intra-arterial infusion (224). However, administering large numbers of magnetic particles into cerebral arteries is associated with a considerable risk of embolization (225). The precision of the approach can be increased by modifying magnetic field topography, thereby alleviating the above-mentioned risk of embolism (166). Further, the cationized model protein β-galactosidase (representing targeted protein drugs such as tumor suppressor proteins and anti-neoplastic enzymes) loaded onto heparin-coated iron-oxide nanoparticles could be precisely deposited in glioma tissue by intra-arterial infusion under MRI surveillance. This also allowed for optimization of magnetic field topography and maintained physiological arterial fluid dynamics, thus representing an effective approach for precise molecular treatment of brain tumors (167). In terms of cell transplantation, MRI enables real-time tracking of bone marrow-derived human mesenchymal stem cells (MSCs) labeled with superparamagnetic nanoparticles (216) or human MSCs expressing ferritin heavy chains (215) in experimental animals (Table 4). In addition, MRI may help to avoid adverse ischemic events in the clinical treatment of glioblastoma multiforme by intra-arterial infusion of tumor-infiltrating lymphocytes. This notion is supported by preclinical data showing that MRI is well-suited to evaluate ischemic events during intra-arterial infusion of activated T cells into the native rabbit brain (226).

#### Other Approaches

There are a number of other approaches to improve intra-arterial administration. The first approach is the combination of compounds to enhance the efficacy of chemotherapeutic drugs. For instance, it was shown that the combination of oral 2% D, L-alpha-difluoromethylornithine and intra-arterial injection of BCNU doubled the median survival time of T9 gliosarcoma-bearing rats by reducing polyamine metabolism required for tumor growth and enhancing anti-tumor cytotoxicity of BCNU (227). Spirohydantoin mustard (spiromustine) promotes BBB penetration and cisplatin deposition in glioblastoma multiforme by combining the anti-tumor reactive moiety of cisplatin with the hydantoin part of spiromustine (228). However, the neurotoxicity of these compounds hampers the interest in further research.

The second approach is the combination of multiple therapeutic regimens. For example, intra-carotid administration of cisplatin combined with intravenous doxorubicin injection is an alternative treatment for patients with inoperable meningiomas (229). Short-term intra-arterial and intravenous chemotherapy prior to radiation increases survival of adult patients with astrocytomas (230). Hyperthermia enhances the cytotoxic effects and deposition of anti-cancer drugs to the tumors. Both preclinical and clinical studies showed that ACNU (231) or adriamycin (232, 233) delivery in combination with local or interstitial brain hyperthermia was associated with higher survival rates. Moreover, intra-arterial administration of carboplatin and melphalan combined with intrathecal topotecan chemotherapy showed a good alleviation effect on a patient suffering from extraocular retinoblastoma with CNS involvement (234). Of note, such combined therapy is often based on individualized treatments. This requires consideration of various factors such as radio- or chemotherapy sensitivity of the tumors, invasion area, and metastatic pathways.

The third approach is the use of new therapeutic agents. Injection of bromodeoxyuridine (anaerobic radiosensitizer) into the external carotid artery through a catheter has been found to increase susceptibility of glioma cells to radiotherapy and increases survival time of treated patients (235). Moreover, intra-carotid injection of recombinant human TNF and lymphotoxin produces remarkable anti-tumor effects in C6 and T9 gliomas-bearing animal models (236). Clinically, intra-arterial administration of TNF-alpha improves the neurological symptoms in patients with glioblastoma multiforme or malignant astrocytomas by inducing coagulation necrosis in central tumor tissue and its feeding arteries (237). With the same strategy, advanced cytotoxins consisting of IL-13, IL-4 or transforming growth factor-alpha have been developed to target glioblastomas expressing these receptors (144, 238, 239). In general, data regarding the efficacy of these cytotoxins is less encouraging when applied in solid tumors. This may be due to the difficulties of large molecules to penetrate the tumor mass, and lower or more variable receptor expression in solid neoplasms.

The fourth approach is gene therapy. Intra-arterial infusion of a p53-containing adenoviral vector can delay the growth of Gli36 glioblastoma tissue carrying a missense-mutated p53 gene (240). Phosphorothioate oligodeoxynucleosides injected intra-arterially were precisely delivered to tumor tissue after BBB opening with bradykinin (241). Recently, intra-arterial infusion of a plasmid encoding an anti-angiogenic endostatin was shown to prolong survival time in the rat 9L gliosarcoma model by decreasing tumor vascular density, perfusion, and permeability (242). Moreover, G47Delta viruses (herpes simplex virus vector with oncolytic replication-competency) carrying deletions of the gamma34.5 and alpha47 genes (243) or the CLN2 gene (244) were injected into the carotid artery after mannitol-mediated BBB opening and had a positive impact on survival in a nude mouse model of cerebral breast cancer metastasis. A recent Phase II clinical trial showed that intra-arterial administration of ganciclovir combined with replication-deficient adenovirus mutant thymidine kinase is effective in improving 6-month progression-free survival, overall progression-free survival, and overall survival in patients with recurrent high-grade gliomas (anaplastic gliomas and glioblastomas) (245). Compared with intratumoral or intracerebral injections in current clinical trials (246, 247), intra-arterial gene therapy expands the treatment area and maximizes the therapeutic effect, but it also puts forward higher requirements for transfection efficiency.

The fifth approach is cell therapy that may benefit from intra-arterial administration. Intra-carotid rather than intravenous administration of a human cytotoxic T-cell line (TALL-104) in the 9L glioblastoma model and a metastatic xenograft model of epidermoid carcinoma enhanced specific anti-tumor effects and significantly prolonged survival time (248). Injection of a murine colon cancer cell line (CT-26) overexpressing interleukin-4 (IL-4) or hemagglutinin antigen can serve as a pre-immunization strategy to prevent metastases from cecum, liver, and lung. Intra-carotid but not systemic administration of CT-26 expressing IL-4 or hemagglutinin antigen is effective to prevent the growth of blood-borne brain metastases (249). In addition, cells may be good “vectors” to be used in intravascular gene therapy. Human MSCs carrying Delta24-RGD oncolytic adenoviruses and labeled with green fluorescent protein are selectively planted into glioma xenografts and exert a strong anti-tumor effect (250). However, it is still too early to translate these approaches into clinical applications.

## Conclusions

**Figure 2:**
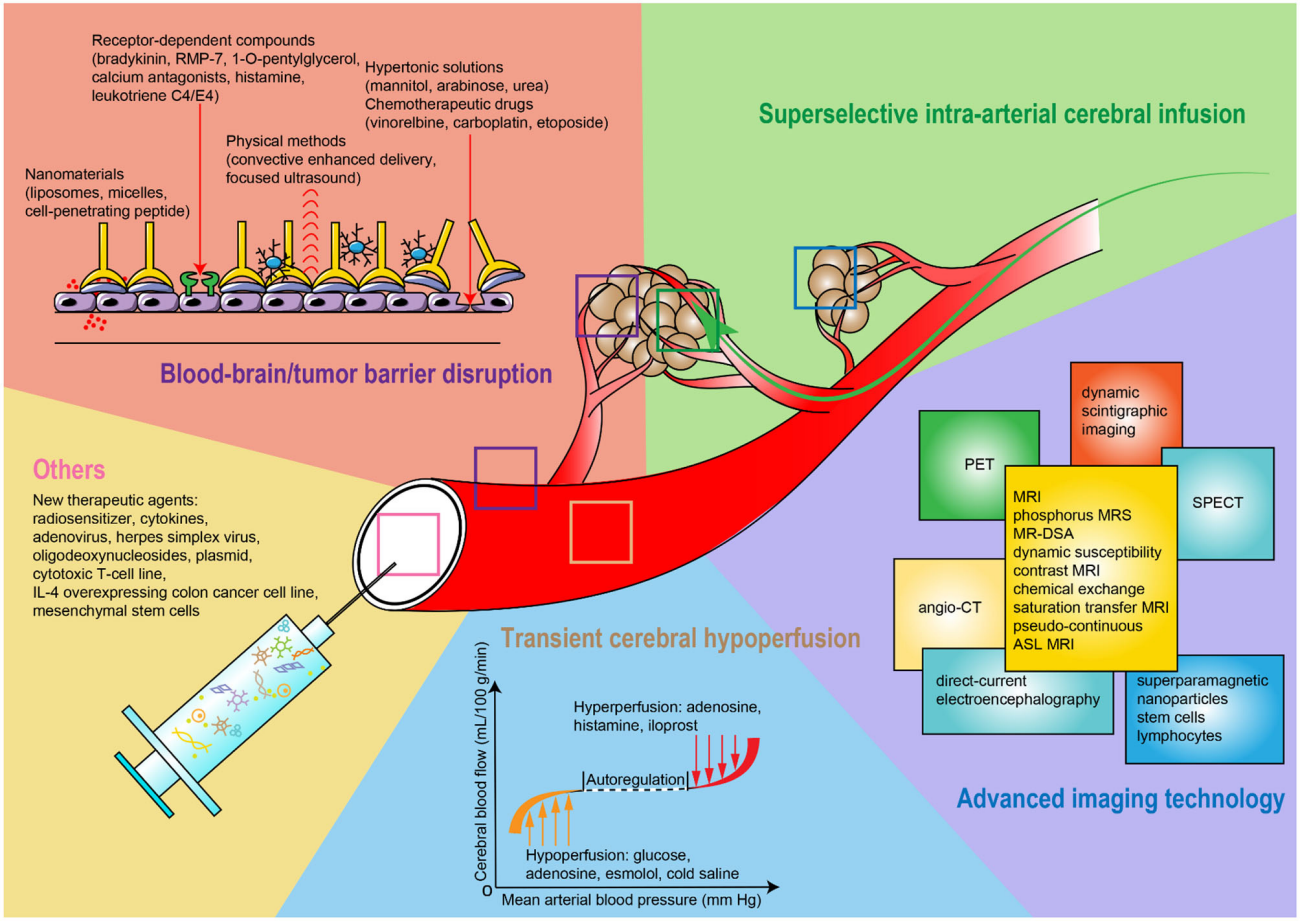
Schematic diagram of strategies for improving intra-arterial administration.

This systematic review comprehensively describes existing strategies for promoting the accuracy of intra-arterial drug delivery in experimental and clinical brain tumor therapy (Figure 2). These strategies show a promising potential to innovate and optimize many aspects of brain tumor treatments, including the accurate prediction of targeted tumor tissue, the effectiveness of drug transfer over the BBB/BTB, and the precise deposition of the therapeutic agent(s) in the tumor. Moreover, methodological advances foster the development of multidisciplinary treatment strategies that can result in better treatment effects and might represent a promising way forward in the treatment of brain tumors. However, translating new and improved methods from animal experiments to clinical practice still has to face many challenges. First, genetic, molecular, immune, and cellular differences between humans and other species prevent animal models from replicating the whole spectrum of important aspects of human pathophysiology under disease-specific conditions. Second, experimental animals exhibit different tolerances to drug toxicity, which makes it difficult to accurately assess the safety and effectiveness of therapeutic or adjuvant drugs before applying them to patients. Third, the number of patients included in early-stage clinical trials is usually insufficient to reveal all but the strongest effects, while patient age, sex, tumor type, course of disease, and adjunct treatment strategies are difficult to standardize or even harmonize. This limits the predictive value of obtained results. Based on this, new disease models have attracted much interest. The “tumor graft models” or “patient-derived xenograft” models are valuable humanized models to maintain the tumor heterogeneity and genetic characteristics of the patients by implanting tumor tissues removed from surgery into immunodeficient mice. Another breakthrough is the development of human brain organoids, which can be produced by patient-derived tumor cells or tissues in a 3D *in-vitro* culture system. These organoids have greater feasibility in large-scale screening of therapeutic agents and can exhibit their aggressiveness and proliferative ability and establish vascular system in host brains. In addition, some large animals exhibit an anatomical structure of the brain and its blood vessels being similar to that of humans, which may be a good option to optimize intra-arterial procedures. These models can also make excellent use of clinical imaging technologies (251). Taken together, this forms an excellent basis to further refine intra-arterial approaches for the treatment of brain tumors to ultimately improve clinical treatment regimens.

## Data Availability Statement

All datasets generated and analyzed for this study are included in the article/supplementary material.

## Author Contributions

RH, JB, and SL defined the search strategy, conducted the literature search and review, analyzed the data, and drafted the manuscript. All authors approved the final manuscript version.

## Conflict of Interest

The authors declare that the research was conducted in the absence of any commercial or financial relationships that could be construed as a potential conflict of interest.
